# Breakfast choice: An experiment combining a nutritional training workshop targeting adolescents and the promotion of unhealthy products

**DOI:** 10.1002/hec.3549

**Published:** 2017-07-25

**Authors:** Toni Mora, Beatriz G. Lopez‐Valcarcel

**Affiliations:** ^1^ Universitat Internacional de Catalunya Barcelona Spain; ^2^ Universidad de Las Palmas de Gran Canaria Las Palmas Spain

**Keywords:** nutrition programme, randomised control trial, unhealthy promotion

## Abstract

A randomised control trial was conducted to determine changes in the food and drink choices of adolescents following their participation in a 50‐min nutrition workshop. The experiment was conducted at 104 schools in Barcelona (126 classes, 3,291 adolescents). Schools were randomly selected and stratified by district and by public or private status. The students were given three types of vouchers with different options regarding the type of food for which the vouchers could be exchanged (standard for healthy food and drink, two for one for unhealthy food, and two for one for unhealthy drink). Difference‐in‐differences linear models that control for individual, family, school or neighbourhood characteristics, and the influence of peers were applied. The probability of students' choosing unhealthy food and drink fell by 7.1% and 4.4%, respectively, following participation in the nutrition workshop. The promotion of unhealthy beverages counteracted the positive impact of the workshop on beverage choice.

## 1 INTRODUCTION

As observed by Cash and McAlister (2013), children comprise a primary market (their own purchases), an influence market (affecting parental spending), and a future market (when they become adult customers). With regard to these present and future customers, making a change towards healthier eating and lifestyle habits is known to be extremely difficult in adulthood (Hill, 2009), and it is well established that the health status acquired during childhood and adolescence has a major influence on the patterns of adult life (see Almond & Currie, 2011, for an extensive review). Finally, rationality only becomes significantly present in early adolescence (Harbaugh, Krause, & Berry, 2001). These considerations amply justify investing resources in programmes to influence adolescents' food and beverage choices. A great deal of research has been conducted regarding optimising or attempting to optimise adolescents' dietary choices. In the field of health economics, health economists have explored improving children's and adolescents' food choices by randomised control trials focussing on the provision of incentives and evaluating educational nutrition workshops.

Although a large public policy effort was devoted to investigating the impact of nutrition education on consumption (see Ruel & Alderman, 2013, for a review), in real life, adolescents are exposed to marketing and discounts by manufacturers to encourage more consumption of unhealthy foods. Those stimuli may counteract the effects of healthy nutrition programmes. Nevertheless, there are no studies evaluating the impact of nutrition education in an environment characterised by marketing efforts and promoting the consumption of unhealthy food that may counteract the beneficial effects of the educational programmes.

One of our primary contributions in this paper is filling that gap by combining an educational nutrition programme with the promotion of unhealthy food and drinks (two‐for‐one deals) to emulate the price discounts operating in real markets. We estimated the impact of a short nutrition workshop on 13‐year‐old adolescents' food and drink choices (healthy vs. unhealthy) for their midmorning snack at school. In doing so, we distinguished between the two types of consumption (food and drink) to determine whether nutrition workshops are equally effective for different products. To the best of our knowledge, this is the first study to make this comparison. Then, we combined this nutrition workshop with a price discount offer to randomly chosen students. We analysed the extent to which the students reacted to the promotion of unhealthy products. Unlike previous studies in which rewards were offered for making healthier choices or in which healthy foods such as vegetables were promoted at school‐based interventions, our intention in this trial was to examine how teenagers reacted to a two‐for‐one offer of unhealthy nutrition choices. To some extent, this analysis reflects adolescents' reactions to the commercial marketing practices that promote unhealthy choices. In this trial, an aspect of fundamental importance is that we distinguished between food and drink in our analysis of whether the promotion of less healthy products is counteracted by participation in an educational workshop. In addition, we calculated the price sensitivity of young people to various food and drink options. Again, to the best of our knowledge, no previous study has focussed on these factors.

In addition to the above, it is significant that this study of adolescents' nutrition preferences was conducted in a large European city (Barcelona, Spain). The randomised selection of the schools was stratified using proportionate stratification by city district and by their public or private status. We had access to a large sample size. The experiment was conducted at 104 schools, in 126 classes and with the participation of 3,291 adolescents. Three types of food and drink vouchers were provided, with options favouring the less healthy nutritional choices (standard, two‐for‐one unhealthy food, and two‐for‐one unhealthy drink).

In our experiment, the study subjects (in both the experimental group and the control group) were asked to choose from among four alternative foods and four types of drinks for their midmorning snack. We brought the selected items to the school within the following two school days. Two of the foods and drinks were defined as *healthy* by the project's nutritionist consultant, and the other two were not. The midmorning snack was selected for analysis instead of the lunch because the content of the latter, when provided at school, is fully regulated in Spain. Unlike in other cultures, lunch in Spain comprises two courses plus dessert and is the main meal every day (Aranceta, Pérez, Serra, & Delgado, 2004). Students generally have breakfast at home before attending school, and then after 2 h of lectures, they stop and have their midmorning snack that they bring from home, which is generally a sandwich and sometimes complemented by a drink. In advance, we announced to the tutors that we were going to provide the snack meal so that the pupils would abstain from bringing their own snacks from home on the scheduled day.

Under the theory of random utility (McFadden, 1986) and assuming that adolescents are rational agents, the students will choose the option that maximises expected utility, constrained by the feasible choice set. Expected utility basically depends on the attributes of each option available, on the students' tastes, and on the cost of the items. The nutrition workshop was designed to change the students' perceptions of the attribute in question (the healthiness of the food or drink) and the weight assigned to that attribute. The promotion of unhealthy items was designed to allow us to evaluate the counteracting effect of the price by changing the feasible set of choices (constraints in the maximisation process).

In summary, the following results were obtained.

1. The adolescents' choices of food and drink were fairly heterogeneous. At baseline, half of the participants chose an unhealthy food, and approximately two thirds chose an unhealthy drink, generally cola.
2. The training workshop was effective in promoting changes towards healthier options, more so for food than for drink. Thirty‐one per cent of the students in the experimental group switched to a healthier food option, compared with 20.2% in the control group. However, the corresponding percentages were significantly lower for drinks (19% in the experimental group and 11.6% in the control group). The econometric model corroborated this descriptive result, reflecting a reduction of 7.1% in the number of students choosing an unhealthy food and a reduction of 4.4% in those opting for an unhealthy drink.
3. The students were sensitive to price (as represented in the two‐for‐one promotion of unhealthy products) in the second round of the trial. In contrast to the outcome for the nutrition workshop, the effect of price promotion was greater for drinks than for foods.
4. The adolescents' peers exerted a large and statistically significant impact on their choices.

The rest of this paper is organised as follows. Section 2 discusses the background and the previous research in this field. Section 3 describes the experimental design and the econometric strategy applied. Section 4 presents the empirical results obtained, and finally, Section 5 discusses these findings, considering their implications and acknowledging the study limitations.

## 2 CONCEPTUAL FRAMEWORK AND RELATED LITERATURE

Many studies have analysed dietary choices among children and adolescents and have evaluated interventions focussing on these choices (see Cash & McAlister, 2013, for a review). Reducing the price of healthy products or taxing unhealthy ones may affect adolescents and adults in different manners, depending on the extent to which the tax is transferred to the retail price and on the price elasticity of demand among the population in question. This question is directly relevant to the effectiveness of nutrition interventions but has received little research attention. In this study, an in‐depth analysis is performed in this respect, with particular emphasis on determining the outcome when less healthy food and drink products are promoted. By contrast, previous researchers have focussed on promoting healthier products via price reductions (French et al., 1997) or by means of randomised control trials by offering rewards or incentives (Belot, James, & Nolen, 2013; Just & Price, 2013; List & Samek, 2015; Loewenstein, Price, & Volpp, 2016) or by providing personalised advice to persuade consumers to switch to healthier options (Bedard & Kuhn, 2015). Addressing the question of the impact of providing education, the field experiment conducted by List and Samek (2015) was based on a sample of low‐income children in the Chicago area, and the incentive offered was a small‐value prize for the children in the experimental group who chose the healthy option. The study goal was to compare incentives, designed as losses versus gains. The authors concluded that educational messages are most effective when combined with a small individual incentive. The results of their experiment indicated that interventions based on a combination of messages and incentives may be quite effective in promoting the choice to consume healthy foods and, moreover, that the effect is a lasting one. Another interesting field experiment with more than 1,400 schoolchildren in Chicago demonstrated the impact of interventions based on two behavioural theories and demonstrated that the health information provided by teachers to children is effective in moving the latter to make healthier choices (Samek, 2016). A problem is that the effects of extrinsic incentives may disappear once the incentive is removed.

We addressed a tactic that is commonly adopted by supermarkets and corner stores, that is, the promotion of unhealthy food and drink by means of big discounts on second purchases and vouchers. What would occur if the price paid by children and adolescents for unhealthy food and drink products, such as cola, were reduced (by manufacturers or retailers)? Cawley and Frisvold (2015) reported that retail prices rose by less than half of the amount of the sugar‐sweetened beverage tax introduced in Berkeley (California) and concluded that manufacturers and/or retailers were absorbing the effect of taxation on consumers.

Solomon, Russell‐Bennett, and Previte (2012) analysed the process by which children grow up and mature, concluding that by the age of 12, many children have become, or are about to become, autonomous consumers. Solomon et al. considered children's behaviour and their role as economic agents making their own purchasing decisions. Children are heterogeneous in their preferences in terms of cognitive skills and maturity, risk aversion, and intertemporal discount rates (Harbaugh, Krause, & Vesterlund, 2002; Steinberg et al., 2009). McClain, Chappuis, Nguyen‐Rodriguez, Yaroch, and Spruijt‐Metz (2009) reviewed the psychosocial factors that influence adolescents' eating behaviours, according to the literature, and observed that knowledge, tastes, and norms are strong influences. In addition to the unobservable individual heterogeneity that may influence their tastes and choices, it is plausible, and an empirical matter, that some individual heterogeneity is associated with certain observable individual characteristics, such as gender, age, and parental socio‐economic status. The following empirical questions then arise: Are less wealthy adolescents (represented by the proxy *parents' level of education*) more likely to prefer less healthy products? (Hallström et al., 2011; Ranjit et al., 2015). Do more highly skilled students make different choices from their less skilled counterparts? The literature suggests that academic performance is a predictor of healthier eating habits (Fu, Cheng, Tu, & Pan, 2007). Are choices determined by maturity? In this regard, Harbaugh et al. (2001) noted that children aged 11 years behave as rationally as adults when making choices, although this question is linked, to some degree, to their skill levels. Do students at publicly funded schools react differently from those educated privately? Do girls behave in the same manner as boys? Among British schoolchildren, preferences towards healthy foods (fruits and vegetables) are more pronounced for girls than for boys, and the opposite is true for preferences for fatty and sugary foods and processed meat products (Cooke and Wardle, 2005). All of these questions are addressed empirically in our econometric study.

## 3 EXPERIMENTAL DESIGN

### 3.1 Experimental environment

**Figure 1:**
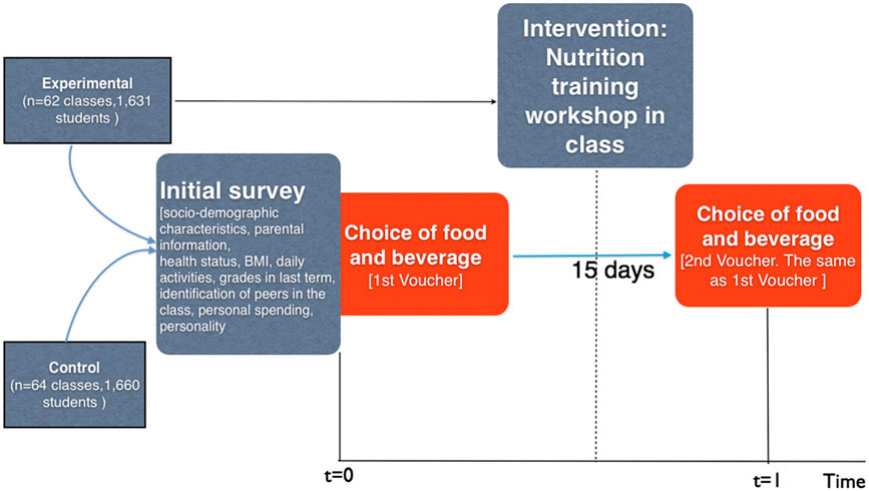
Experimental design. BMI = body mass index [Colour figure can be viewed at http://wileyonlinelibrary.com]

The experiment was conducted at 104 schools in Barcelona (Spain) in 126 classes and with the participation of 3,291 seventh‐grade students (aged 12–13 years). In Barcelona, 213 schools provide secondary education (64 public and 149 private). We used seventh graders in our study primarily because they are nearing the age to make their own choices and developing rationality and it is important for policy to promote positive long‐term outcomes rather than adolescents' short‐term goals (Reyna & Farley, 2006). We used proportionate stratification of schools by district (of which Barcelona has 10, with varying socio‐economic characteristics) and public or private status. Schools were randomly selected based on the abovementioned strata. Then, classrooms in the selected schools were randomly assigned to a control or experimental group in the same proportion. If a school declined to participate, another school with the same characteristics (district, size, and ownership) replaced it. We believe we were not affected by selection bias although it is a limitation that cannot be tested because additional information is not publicly available in the Catalan educational system. In all, 29 of 104 school centres were substitutes. If more than one class was eligible in a specific school (large schools with more than one seventh‐grade class), two classrooms were randomly selected by the researchers; one was assigned to the control group and the other to the treatment group. In this case, both classrooms participated simultaneously to avoid potential spillover effects, that is, to avoid one classroom influencing the other during the recreation time between lectures, when students eat their midmorning snacks. Food and drink choices were selected simultaneously in different classrooms in a given school; thus, students from treatment and control groups did not interact when choosing food and drink. The study occurred between February and April 2015. Figure 1 describes the experimental structure. In preparing this study, initial data were obtained from the Consorci d'Educació de Barcelona (Barcelona Board of Education) regarding district size (students enrolled per district and academic year), the private or public status of the school (ownership), and addresses and contact details (telephone and email).

An initial online survey was conducted, with 30 questions, which took approximately 15 min to complete (see questionnaire in Appendix S1). The students were asked for information regarding their sociodemographic characteristics, parental information (occupational status and level of education), health status, height and weight, daily activities, their most recent term grades in mathematics and in the Spanish language, whether they were given pocket money, and what type of food and drink they spent it on (sugary drinks, sandwiches, etc.) in their free time. In Spain, a list of students is generated in each class in alphabetical order by family name; each student name is assigned a number. This class number list was presented during the conducting of the survey because the students were asked on the questionnaire to indicate their peers (friends) in the class according to this list. There was no limit to the number of friends who could be designated. Thus, we were able to match the students with their peers because students had previously stated their list number as a password to access the online questionnaire. Finally, the students were asked questions aimed at ascertaining their personality type, following a procedure employed in previous research in this field (Mora & Oreopoulos, 2011).

The final page of the online questionnaire included two questions regarding the students' midmorning snack preferences when they were offered a choice free of charge. Teachers were asked to ensure that students not talk to one another when they answered the questionnaire and when making choices at the end of the survey. Indeed, teachers were surveyed once the control trial ended regarding how the experiment was implemented at their school centre. Two healthy and two unhealthy options were included for food and drink, these options previously having been discussed and defined with a nutritionist. The students were each asked to select one food item and one drink from the following products: for food, a ham sandwich plus sliced fruit (healthy option), a tuna sandwich plus sliced fruit (healthy option), a croissant (unhealthy option), or a cupcake (unhealthy option). The students were also asked if they had any food or drink intolerance. As previously noted, this is a meal that students generally bring from home, and it is commonly a snack. For drinks, the choices offered were mineral water (healthy option), milk (healthy option), cola (unhealthy option), or a drink that is a combination of milk and sugared fruit juice (unhealthy option). All items were free. All of these choices were suggested by the project's consultant nutritionist (the head nutritionist at the Health Promotion of the Catalan Health Department), who also designed the workshop. Table S1 in the supporting information details the nutrition content in grams of the foods and beverages analysed. Unhealthy drinks were determined to have substantially more added sugar than healthy ones. Cola, with 32.5 g of sugar, and mineral water, with 0 g, were the extreme items. The primary nutritional differences in the food analysed were in the content of saturated fatty acids (1.90 and 3.88 g for the healthy food vs. 5.45 and 6.82 g for the unhealthy food) and in the content of fibre (4.2 g for the healthy options vs. 1.21 and 0.55 g for the unhealthy items). However, according to the nutritionist, there were no significant differences in the calorie content between the healthy and unhealthy foods. Our research was reviewed by an ethics board at the Universitat Internacional de Catalunya, and consent was requested of the Asociaciones de Madres y Padres de Alumnos (parental associations) at each school.

The students' options were associated with a price stimulus, making unhealthy options cheaper for some students. Thus, although all of the offered items were free, the unhealthy products were promoted by means of a two‐for‐one offer to randomly selected students (two unhealthy items or one healthy item). Three types of vouchers were randomly assigned (by the class number list, created in alphabetical order by family name): a standard voucher (numbers 1, 4, 7, …), a voucher enabling the student to obtain two for one of an unhealthy food option but with the standard drink choices (numbers 3, 6, 9, …), and a voucher offering two‐for‐one unhealthy drinks but with the standard food choices (numbers 2, 5, 8, …). The items selected by each student were delivered approximately two days later, appropriately packaged and marked with each student's number from the list. Each student received a second meal voucher, exactly the same as the first one, 15 days after completing the survey; however, half of the students, those in the experimental group, attended a nutrition workshop during this 2‐week period. For those in the treated group, nutritionists monitoring the nutrition workshop provided the vouchers at the end of the session. For those classrooms in the control group, we provided the tutors with the vouchers that they should give to the students 15 days after completing the survey. Again, the selected items were delivered 2 days after selection. Students were not observed when the food and drink were provided; thus, we did not know whether they traded with one another. We knew only their choices, not their final consumption. With regard to the possible presence of experimenter bias because choices were made in front of the nutritionists or the tutors, we asked the adults to display the food and indicate that students were free to choose. In all cases, tutors were responsible for providing the vouchers to students and clarifying how students should proceed. Nutritionists were asked to remain outside the classroom during the selection process, which is a limitation of the study.

The nutrition workshop that was conducted in the treated classrooms was designed by the Head of Nutrition at the Health Promotion of the Catalan Health Department. Three nutritionists were trained by the head nutritionist to lead the workshops in the various classes in the treatment group. The workshop was a game called “You Know, You Choose,” and its duration was 50 min (the time of a regular class). Attendance was compulsory because the workshop was scheduled during class time. The game comprised 10 questions with multiple responses, and students participated in teams of up to six people based on the location of their seats (this was determined by tutors) or their order on the classroom list (following the abovementioned alphabetical order). The game was a competition between the teams regarding their nutritional knowledge. To add liveliness to the game, the nutritionists who monitored the 50‐min sessions distributed stickers on which the students wrote their names. Teams were awarded nutritional points for successful answers, and there were rebound answers for the next team when the first team was wrong. Team members had to cooperate and reach consensus on their answer, which was then voiced by the team leader. The content of the workshop was designed to provide information regarding breakfast meals and midmorning meals, sugar and fat content, the relevance of food richer in fibre, and the health‐related problems arising from inappropriate consumption. Slightly more emphasis was placed on drinks than on food. Considering that the standard approach in Catalan schools is an overview of the food pyramid, the questions addressed in the provided workshop referred specifically to the following information: (a) the need to hydrate with water; (b) prioritising lunches and snacks based on the Mediterranean diet (whole‐grain bread, fresh fruit, nuts, milk, yoghurt, olive oil, etc.); (c) prioritising fresh fruit juices and being careful with sugar‐sweetened beverages; (d) lunches and snacks preferably being homemade; (e) the importance of having four or five meals a day; (f) knowing which foods are greasy, fatty, or sugary; (g) the benefits of fibre‐rich foods; and (h) awareness of the relation between excessive fat and sugar intake and health problems, such as tooth decay, obesity, constipation, and a lack of vitamins and antioxidants.

### 3.2 Econometric identification strategy

The following empirical model was considered:

(1) $$y_{\mathrm{ijt}}=\alpha_{0}+\mathrm{\gamma t}+\delta E_{j}+\mathrm{\eta t}E_{j}+x_{\mathrm{it}}^{\prime }\beta +z_{i}^{\prime }\phi +\lambda P_{i}+\mathrm{\psi t}P_{i}+\alpha_{i}+\alpha_{j}+\varepsilon_{\mathrm{ijt}},$$

where *y*_*ijt*_ = 0 if student *i* attending school *j* chooses the unhealthy option at time *t* (*t* = 0 before the intervention and *t* = 1 after the intervention). *E* is a dummy variable that equals 1 for the experimental group, and $x_{\mathrm{it}}^{\prime }$ includes the individual characteristics changing over time, including the percentage of peers who choose the unhealthy food or drink. *P* is the relative price of the unhealthy food or drink (equal to 1 if the price is promoted by a two‐for‐one offer and equal to 0 if the normal price is applied); *α*_*i*_ and *α*_*j*_ are the unobserved individual and school fixed effects, and *ε*_*ijt*_ is the random noise. *ε*_*ijt*_ is assumed to be independent of the explanatory variables. The parameters of interest are *η* (effect of the nutrition workshop) and *λ* (price effect). We estimated Equation (1) by ordinary least squares after applying first differences (within estimate). Standard errors were clustered by schools. Because of the type of experimental design used, the price effect (*λ*) is not identified in Equation (1); however, its change in the second period is identified (*ψ*). The vector *ϕ* of regression coefficients of the *z* variables that do not vary from *t* = 0 to *t* = 1 cancels out in the demeaning (or first differences) transformation. The estimated form of Equation (1) is then

(2) $${\ddot{y}}_{\mathrm{ij}}=\gamma +\eta E_{j}+{\ddot{x}}_{\mathrm{it}}^{\prime }\beta +\psi P_{i}+{\ddot{\varepsilon }}_{\mathrm{ij}},$$

where a diaeresis over a variable indicates the first difference (value at *t* = 1 minus value at *t* = 0). The only *x* variable included in our specification is the peer effect. This variable is measured as the percentage of peers who choose the unhealthy option. Because this parameter may be endogenous, it is instrumented in the robustness analysis following Mora and Gil (2013) with the percentage of unhealthy respondents' friends of friends who are not friends with the respondent, using three‐stage least squares.

To improve estimation efficiency, the two equations in Equation (2) were estimated jointly, thus allowing the ${\ddot{\varepsilon }}_{\mathrm{ij}}$ errors in the food and drink equations to be correlated, as expected in this case because the same unobserved factors that influence preferences for unhealthy food and drink could underlie those determining the overall food and drink choices made by a given student. The standard errors were adjusted by considering within‐group correlation (between observations of the same student). In addition, Zellner's estimator of seemingly unrelated equations was determined; this method is more efficient but assumes homoscedasticity. In our case, because the endogenous is a dummy variable, the model is known to be heteroscedastic. The variances were estimated by bootstrap methods with 2,000 replicates.

To further explore the price effect, we also estimated Equation (2) (omitting *ψP*_*i*_) independently for the subsamples of standard and two‐for‐one vouchers of food and beverages as in Equation (3) and tested the equality of the coefficients. Because the vouchers were selected randomly, the models in Equation (3) are specified with an exogenous sample selection so that *η*_0_ and *η*_1_ can be estimated consistently by ordinary least squares. By testing *η*_0_ = *η*_1_, we determined whether the price promotion of unhealthy food and drinks counteracted the healthy effect of the nutrition workshop.

(3) $$\begin{array}{l} {\ddot{y}}_{\mathrm{ij}}=\gamma_{1}+\eta_{1}E_{j}+{\ddot{x}}_{\mathrm{it}}^{\prime }\beta_{1}+{\ddot{\varepsilon }}_{\mathrm{ij}}\;\text{if}\;P_{i}=1\\ {\ddot{y}}_{\mathrm{ij}}=\gamma_{0}+\eta_{0}E_{j}+{\ddot{x}}_{\mathrm{it}}^{\prime }\beta_{0}+{\ddot{\varepsilon }}_{\mathrm{ij}}\;\text{if}\;P_{i}=0 \end{array}$$

In addition, a number of robustness checks were performed. First, we estimated a disaggregated version of Equation (1), including as endogenous the specific choices among the four alternatives for food and drink and including all of the individual covariates observed in the database. This specification models the primary choices that result from the students' utility maximisation process. Ham sandwich and water were designated as reference categories in the food and drink models, respectively. The specification is a conditional logit model with alternative‐specific covariates (the price). This was estimated for the subsamples unaffected by the nutrition workshop (i.e., the control group at *t* = 0 and *t* = 1 and the experimental group only at *t* = 0). The conditional logit models reflect both the visible sources of the heterogeneity of preferences and the price effect. Although the comparisons made are based on cross‐sectional data, we searched for a systematic relation between the promotion of unhealthy food and drink by price promotion and individual choices.

We also estimated Equation (2) for specific groups of pupils: girls versus boys, low‐income socio‐economic districts, publicly owned schools, receiving pocket money regularly, and less skilled versus more skilled.

## 4 EMPIRICAL RESULTS

### 4.1 Descriptive statistics at baseline

Table 1 shows the primary characteristics of the students assigned to the control group and to the experimental group. On average, the students were approximately 13 years old, and 52% were female. In the study, 15% were overweight or obese, 35% attended public schools (in Barcelona as a whole, the corresponding value is 32.3%), and 7% reported an intolerance to certain foods or drinks. In 95% of the cases, at least one parent was employed. On aggregate, both students' parents jointly had approximately 28 years of education, and 15% of the students had at least one immigrant parent. Finally, 62% of the students received pocket money from their parents. No statistically significant differences in socio‐economic characteristics were observed between the control and treated groups at the 5% significance level except for the variable representing the father's education.

**Table 1:** Baseline descriptive statistics by control and experimental groups: main covariates and outcomes

	Regular voucher		Drink promoted		Food promoted	
	Control group	Experimental group	Control group	Experimental group	Control group	Experimental group
Age	12.81 (0.50)	12.82 (0.49)	12.85 (0.50)	12.84 (0.46)	12.84 (0.50)	12.86 (0.51)
Female	0.54 (0.50)	0.49 (0.50)*	0.50 (0.50)	0.50 (0.50)	0.54 (0.50)	0.50 (0.50)
Immigrant background of at least one parent	0.16 (0.36)	0.16 (0.37)	0.14 (0.34)	0.13 (0.34)	0.15 (0.36)	0.14 (0.35)
Fair or poor health	0.10 (0.30)	0.12 (0.33)	0.09 (0.30)	0.11 (0.32)	0.10 (0.31)	0.12 (0.32)
Overweight–obese	0.15 (0.36)	0.17 (0.37)	0.15 (0.36)	0.16 (0.37)	0.15 (0.36)	0.16 (0.37)
Food–drink intolerance	0.06 (0.23)	0.07 (0.26)	0.06 (0.24)	0.06 (0.24)	0.08 (0.26)	0.07 (0.26)
Extracurricular activity	0.66 (0.47)	0.70 (0.46)	0.70 (0.46)	0.69 (0.46)	0.64 (0.48)	0.67 (0.47)
Father—only able to read and write	0.02 (0.14)	0.02 (0.14)	0.02 (0.14)	0.02 (0.13)	0.02 (0.14)	0.01 (0.11)
Father—primary studies	0.06 (0.24)	0.05 (0.23)	0.06 (0.25)	0.05 (0.22)	0.06 (0.23)	0.07 (0.25)
Father—secondary studies	0.22 (0.42)	0.19 (0.39)	0.20 (0.40)	0.17 (0.37)	0.22 (0.42)	0.20 (0.40)
Father—university studies	0.36 (0.48)	0.41 (0.49)*	0.36 (0.48)	0.40 (0.49)	0.37 (0.48)	0.38 (0.49)
Mother—only able to read and write	0.02 (0.13)	0.02 (0.14)	0.02 (0.15)	0.02 (0.13)	0.02 (0.13)	0.01 (0.11)
Mother—primary studies	0.07 (0.26)	0.04 (0.20)	0.05 (0.22)	0.04 (0.20)	0.05 (0.22)	0.07 (0.26)
Mother—secondary studies	0.22 (0.41)	0.21 (0.41)**	0.22 (0.41)	0.17 (0.38)*	0.23 (0.42)	0.20 (0.40)
Mother—university studies	0.43 (0.50)	0.46 (0.50)	0.41 (0.50)	0.46 (0.50)	0.41 (0.49)	0.42 (0.50)
At least one parent is employed	0.96 (0.20)	0.96 (0.20)	0.95 (0.22)	0.97 (0.17)	0.95 (0.21)	0.95 (0.21)
At least one parent is an immigrant	0.30 (0.46)	0.31 (0.46)	0.30 (0.46)	0.29 (0.46)	0.31 (0.46)	0.32 (0.47)
Weekly pocket money received	0.64 (0.48)	0.64 (0.48)	0.59 (0.49)	0.58 (0.49)	0.59 (0.49)	0.64 (0.48)
Public school	0.32 (0.47)	0.37 (0.48)*	0.33 (0.47)	0.36 (0.48)	0.33 (0.47)	0.36 (0.48)
Chose unhealthy food at *t* = 0	0.48 (0.50)	0.46 (0.50)	0.48 (0.50)	0.45 (0.50)	0.47 (0.50)	0.49 (0.50)
Chose unhealthy drink at *t* = 0	0.74 (0.44)	0.78 (0.42)	0.75 (0.43)	0.76 (0.43)	0.71 (0.45)	0.77 (0.42)**
Statistics (*p* value) of the overidentification test for covariate balance (balance test)	10.96 (0.61)		4.33 (0.99)		2.45 (0.99)	

*Note*. The numbers of students were as follows: 1,129 (regular voucher), 1,100 (drink promoted), and 1,062 (food promoted).

** Statistical significance at 5%.

* Statistical significance at 10%.

There were small differences between the control and experimental groups with regard to the proportion of adolescents who selected unhealthy drinks. Unhealthy preferences were notably stronger for drinks than for food; thus, 47–48% of students chose an unhealthy food product, a figure that rose to 75% for drinks (74% in the experimental group and 77% in the control group). For food, the healthy or unhealthy choice was fairly balanced in all city districts, with 43–52% of students opting for the unhealthy item; however, for drinks, the corresponding range was 67–83% (see Table 2). The latter pattern of preferences is also related to the high‐/low‐income pattern of city districts, which is in turn related to the percentage of state‐funded schools in these areas (note that the public or private status of the schools was one of the stratification criteria applied to the districts).

**Table 2:** Percentages of unhealthy choices across districts in choices at baseline (first voucher)

	Districts	% unhealthy food			% unhealthy drink		
		Proportion	*SD*	*n*	Proportion	*SD*	*n*
Low‐income districts	Nou Barris (68.5)	0.44	0.50	300	0.83	0.38	300
	Ciutat Vella (72)	0.43	0.50	157	0.83	0.38	157
Medium‐income districts	Sants‐Montjuïc (80.7)	0.45	0.50	313	0.77	0.42	313
	Sant Andreu (82.4)	0.51	0.50	248	0.71	0.46	248
	Horta‐Guinardó (85.5)	0.46	0.50	368	0.79	0.41	368
	Sant Martí (85.7)	0.51	0.50	439	0.76	0.43	439
	Gràcia (104.5)	0.49	0.50	269	0.67	0.47	269
High‐income districts	Eixample (116.3)	0.50	0.50	407	0.74	0.44	407
	Les Corts (139.4)	0.52	0.50	206	0.69	0.46	206
	Sarrià‐Sant Gervasi (178.8)	0.43	0.49	581	0.75	0.43	581

*Note*. Districts were categorised according to municipal statistical publications and are presented as ranked based on per capita disposable income, expressed in parentheses following the district name (Barcelona = 100 in 2005). Analysis of variance *F* test (*F* = 1.73, *p* = .08) did not reject the equality of percentage of unhealthy food (5% significance) among districts and rejects the null hypothesis for percentage of unhealthy drink (*F* = 3.83, *p* = .00).

Table 3 shows the food and drink choices made at baseline, depending on the type of voucher (standard or one of the two‐for‐one variants) received. The three types were distributed equally; thus, each type was received by one third of the students. With respect to the less healthy options, the students' preferences varied for food and drink. Whereas 47.1% of the students chose either a croissant or a cupcake, 75.4% chose one of the unhealthy drinks. A notable finding was that the most commonly selected items were the croissant and the ham sandwich plus sliced fruit (each chosen by 37%) and the cola drink (49%). Nevertheless, the students' initial choices did not appear to be driven by the type of voucher received because the percentages for each choice were distributed nearly equally across the different vouchers, and the statistical test did not reject independence. In Table S2 of Appendix S3, we report the bivariate probit coefficients of price promotion at baseline (*t* = 0) and their standard errors corrected with bootstrap (250 replications). As seen, at baseline, the price effect, although positive, was not statistically significant.

**Table 3:** Percentages of food and beverage choices based on the kind of voucher at baseline

	Standard	Promoting unhealthy food	Promoting unhealthy beverage	Total % selection
Ham sandwich plus sliced fruit	36.0	36.1	38.8	37.0
Tuna sandwich plus sliced fruit	17.0	15.7	14.9	15.9
Croissant	36.8	38.8	36.6	37.4
Cupcake	10.2	9.4	9.7	9.7
	100	100	100	100
χ^2^ test (*p* value)	4.28 (.64)			
Mineral water	17.4	19.4	18.9	18.5
Milk	6.6	6.0	5.5	6.1
Milk plus fruit juice	27.7	26.1	26.3	26.7
Cola	48.3	48.5	49.3	48.7
	100	100	100	100
χ^2^ test (*p* value)	3.22 (.78)			

*Note*. Cell percentages are shown. Pearson's chi‐squared contingency test did not reject the null hypothesis of independence.

### 4.2 Transitions

Table 4 presents the results for transitions (changes in preferences), presenting the students' first and second choices and splitting the sample into control and experimental groups. Those in the experimental group were more likely to transit to healthy options and less likely to transit from healthy to less healthy options. In both groups, rates of transition between healthy and unhealthy choices were higher for foods than for drinks. Specifically, 31% of the students in the experimental group switched to a healthier food option compared with 20.2% in the control group; these percentages were significantly lower for drinks (19% and 11.6%, respectively). Conversely, 31% of students in the experimental group switched to an unhealthy food option compared with 38.4% of those in the control group (the corresponding values for drinks were 44.2% and 56.2%).

**Table 4:** Transitions between choices by the control and experimental groups

	Control group		Experimental group		Control group		Experimental group	
	Healthy food *t* = 1	Unhealthy food *t* = 1	Healthy food *t* = 1	Unhealthy food *t* = 1	Healthy beverage *t* = 1	Unhealthy beverage *t* = 1	Healthy beverage *t* = 1	Unhealthy beverage *t* = 1
Healthy food *t* = 0	61.6	38.4	69.0	31.0				
Unhealthy food *t* = 0	20.2	79.8	30.5	69.5				
Healthy beverage *t* = 0					43.8	56.2	55.8	44.2
Unhealthy beverage *t* = 0					11.6	88.4	19.0	81.0

The results presented in Table 4 suggest that the nutrition workshop intervention was effective. To control for other variables and to estimate the effect of prices, in the next section, our analysis is extended by means of a multivariate difference‐in‐difference estimation.

### 4.3 Estimation results

Table 5 presents the primary results of the estimation of Equation (2). All coefficients have the expected signs and are significant at 1%, except for the effect of price on food choice following the nutrition intervention, which is only statistically significant at 10%.

**Table 5:** Main results: difference‐in‐difference estimation (Equation (2))

	Unhealthy food choice	Unhealthy beverage choice
Time effect (dummy for second voucher)	−0.047 (0.02)***	−0.111 (0.02)***
Workshop impact	−0.071 (0.01)***	−0.044 (0.01)***
Unhealthy peers impact	0.258 (0.02)***	0.174 (0.03)***
Two‐for‐one promotion at *t* = 1	0.030 (0.02)*	0.041 (0.01)***
*N*	3,264	3,264
*R* ^2^	.0381	.0124
χ^2^ (*p* value)	255.56 (.00)	81.12 (.00)
ρ (correlation between errors of the two equations)	.095	
Breusch–Pagan	29.67 (0.00)	

*Note*. All results were obtained by seemingly unrelated equation estimation. Bootstrap analysis was performed, with 2,000 replicates. Standard errors were clustered at the school level.

*** Statistical significance at 1%.

** Statistical significance at 5%.

* Statistical significance at 10%.

The effect of the workshop (*η*) was to reduce by 7.1 percentage points (pp) the probability of unhealthy food being chosen and reduce by 4.4 pp the corresponding probability for drinks. Therefore, as a result of the workshop, the consumption of unhealthy items fell, this reduction being more pronounced for food than for drinks.

At *t* = 1, the nutrition choices were healthier than at baseline for both groups (experimental and control). This pattern was more intense for drinks than for food. However, when preferences for unhealthy items were promoted by reducing their relative price (i.e., offering two for one), the probability of an unhealthy item being chosen at *t* = 1 rose by 3 pp for food and by 4.1 pp for drinks (*ψ*). Thus, the promotion of unhealthy items counteracted the effect of the nutrition workshop, particularly for drinks. Indeed, in the latter case, the coefficients for the effects of the experiment and those of the price change were equal, with opposite signs, implying that the workshop had no effect on students who received a promotion voucher. In the case of food, the nutrition workshop had a net effect of −0.071 − (−0.030) = −0.041 on the students who were offered the promotion voucher.

According to the estimation of Equation (2), the students' peers had a large and statistically significant association with their nutrition choices. Between a student whose peers prefer unhealthy food and drink and another whose peers prefer healthier items, there is a 25.8‐pp difference in the probability of him or her choosing unhealthy food and a difference of 17.4 pp with respect to drinks. To further explore the possibility that the peers are endogenous, we instrumented them (see Appendix S4 for details).

The correlation between the errors of the two equations is 0.095 and statistically significant (Breusch–Pagan test statistic = 29.67, *p* = .00). This result confirms our expectation that there is a relation between healthy and unhealthy preferences for food and drink.

The estimates of Equation (3) corroborate the differences observed between food and drink choices in Equation (2) (Table 6). When estimated separately for standard and promoted food vouchers, the effect of the workshop is the same (the 95% confidence intervals overlap). Therefore, the possibility that *η*_0_ = *η*_1_ cannot be rejected. For drinks, although the respective confidence intervals also overlap, the effect of the workshop intervention is insignificant at 5% for the students given the promoted voucher. The peer effects were also nonsignificant for the students given the two‐for‐one vouchers for drinks, whereas peer influence had an equal effect on those given promoted and nonpromoted food vouchers. In summary, significant differences were observed in the students' preferences concerning food and drinks.

**Table 6:** Main results by kind of voucher

	Unhealthy food choice		Unhealthy beverage choice	
	Regular voucher	Two‐for‐one unhealthy food	Regular voucher	Two‐for‐one unhealthy drink
Time effect	−0.043 (0.02)*	−0.026 (0.03)	−0.144 (0.02)***	0.002 (0.04)
Workshop impact	−0.074 (0.01)***	−0.064 (0.02)***	−0.047 (0.02)**	−0.037 (0.02)*
Unhealthy peers impact	0.253 (0.03)***	0.268 (0.03)***	0.222 (0.02)***	0.070 (0.05)
*N*	2,216	1,048	2,171	1,093
*R* ^2^	.0352	.0438	.0163	.0085
χ^2^ (*p* value)	159.13 (.00)	95.18 (.00)	68.33 (.00)	19.17 (.00)
ρ	.082	.120	.100	.080
Breusch–Pagan	14.82 (0.00)	15.02 (0.00)	21.89 (0.00)	7.04 (0.00)

*Note*. All results were obtained by seemingly unrelated equation estimation. Bootstrap analysis was performed, with 2,000 replicates. Standard errors were clustered at the school level.

*** Statistical significance at 1%.

** Statistical significance at 5%.

* Statistical significance at 10%.

### 4.4 Robustness tests

Table 7 presents the estimates of the price effect (achieved by the promotion of unhealthy items) according to the conditional logit models for cross‐sectional observations unaffected by the experiment (control group and intervention group at baseline). The models, for food and drinks, are adjusted for the individual, household, and environmental covariates that may influence preferences and choices. The models are also adjusted for circumstances in the class that may have influenced the students' choices during the survey, such as the teacher's actions (e.g., in preparation for the survey or in advising the students on their answers) or the fact that students may have interacted while filling out the questionnaire. Another aspect taken into consideration was the elapsed time between distributing the first and second vouchers. The individual and household characteristics included in our analysis were the students' ages, genders, immigrant status, health status, skills, conscientiousness as a personality trait, body mass index, intolerance to specific foods or drinks, regular physical activity, parents' level of education and employment status, country of origin (Spain or elsewhere), and pocket money received from their parents (which is their only source of money because they are below the minimum legal age to work). Also included were dummy variables for attendance at state‐funded rather than private schools and fixed effects for city districts. Standard errors were clustered at the school level. Although the empty model (model without covariates) reveals systematic strong preferences for the cola drink and the croissant food, after covariates are adjusted for, the specific intercepts in the conditional logit equations were no longer significant. It is evident, then, that the preferences are heterogeneous and that some of the characteristics of the students and their environment explain such differences to an extent. The conditional logit model suggests that the price promotion may influence the choice of unhealthy food (the score increased by 0.136, *p* < .00) but not the choice of an unhealthy drink (coefficient = .049 and is statistically nonsignificant).

**Table 7:** Conditional logit model of specific choice of food and beverage: the effect of the promotion of unhealthy items

	Food choice	Beverage choice
Promotion of unhealthy items	0.135 (0.06)***	0.049 (0.08)
*N* (records)	19,788	19,788
*N* (cases)	4,947	4,947
χ^2^ (*p* value)	718.55 (.00)	977.45

*Note*. The base category refers to ham sandwich plus sliced fruit and water for food and beverages, respectively. Regressions controlled by means of age, gender, immigrant status, health status, skills, conscientiousness, body mass index, intolerance to food or drink, regular physical activity, parents' level of education, parents' employment status, parents' immigrant status, pocket money, public school, and teacher's intervention. Standard errors are clustered at the school level.

*** means signifficant at 1%.

We also determined the effects of variables related to conditions regarding the performance of the survey (whether the students conversed while answering or whether the teacher advised them). The teachers were asked to provide this information the day after the experiment ended at their school. None of these variables was determined to be statistically significant after running regressions at a cross‐sectional level for the initial period. However, Table 8 indicates that there were statistically significant differences between the control and experimental groups in two specific situations during the first choice: teachers talking to the students after conducting the survey and the presentation of food and drink selections as an outcome and not a reward for participation in the survey. Note that we specifically asked teachers how they introduced the voucher to their students. In both cases, the percentages were higher for the experimental group.

**Table 8:** Differences in teachers' presentation of the survey: at the class level

	Control group	Experimental group
Teacher prepared survey beforehand*	0.11 (0.32)	0.03 (0.18)
Teacher had a chat with students after the survey**	0.42 (0.50)	0.61 (0.49)
Teacher gave advice about healthy food at *t* = 0	0.21 (0.41)	0.22 (0.42)
The students talked during the survey	0.22 (0.42)	0.11 (0.46)
Food and drink were introduced not as a reward**	0.28 (0.45)	0.47 (0.50)
Time lag between the first delivery of food–drink and second selection	10.13 (6.36)	9.08 (6.97)

** Significance at 5%.

* Statistical significance at 10%.

Finally, the study sample was split according to specific characteristics that may be related to preferences and choices. In this regard, gender was a candidate because most previous studies observed systematic differences between girls and boys (Hallström et al., 2011). Income (receiving pocket money and living in a non‐high‐income district) is another interesting variable as a potential source of differences in preferences and choices. We also considered the impact on overweight children because those children are of the greatest interest to the sample and the primary target of the nutrition interventions. Immigrant status may also be a systematic source of differences. Nevertheless, the immigrant and overweight groups were too small for meaningful analysis (*n* = 477 and *n* = 512, respectively). Accordingly, a regression analysis was not conducted because of the poor power of the tests. Table 9 shows the primary results for different groups of students with regard to the impact of the nutrition workshop. This table only shows results for cases in which the sample was sufficiently large. The impact of the workshop on food and drink choices was significant and approximately the same for girls; noticeably, however, there was no impact on beverage choices for male students. The workshop did not demonstrate any significant impact on the drink choices for students in public schools. Excluding those adolescents in high‐income districts, the estimated impact of the workshop is the same as in the full sample for food and slightly larger for beverages.

**Table 9:** Main results for workshop impact: specific sample groups

Workshop impact coefficients	Unhealthy food choice	Unhealthy beverage choice	Sample (*N*)
Overall coefficient (Table 5)	−0.071 (0.01)***	−0.044 (0.01)***	3,264
Male	−0.075 (0.03)***	−0.010 (0.02)	1,593
Female	−0.066 (0.03)**	−0.077 (0.02)***	1,671
Pocket money received	−0.073 (0.02)***	−0.050 (0.02)**	2,004
Public school	−0.087 (0.03)***	−0.039 (0.028)	1,119
Not a resident of a high‐income district	−0.078 (0.02)***	−0.059 (0.02)***	2,073
Less skilled (below the 25th standardised grade)	−0.091 (0.04)**	−0.050 (0.03)	818
More skilled (above the 75th standardised grade)	−0.077 (0.04)*	−0.002 (0.04)	807

*Note*. All results were obtained by seemingly unrelated equation estimation. Bootstrap analysis was performed, with 2,000 replicates. Standard errors were clustered at the school level.

*** Statistical significance at 1%.

** Statistical significance at 5%.

* Statistical significance at 10%.

## 5 DISCUSSION

This study, novel in its scope, highlights the heterogeneous effects of a school‐based nutrition intervention. The survey results indicated that teenagers' food and drink preferences in different circumstances were consistently different and that they were more reluctant to internalise advice against consuming sugar‐sweetened beverages than to reduce their consumption of unhealthy sugared food. This finding suggests that it is more difficult to reduce the consumption of unhealthy drinks (such as colas) than unhealthy food. In addition, the adolescents in our study groups were observed to be sensitive to a pricing promotion of unhealthier products although this effect was only marginally significant in the case of the less healthy food.

An important reason for the differences in the food or beverage impact is that food cannot be stored whereas beverages can. Hence, two‐for‐one discounts may be more effective for beverages than food items. This could be why the pricing discounts were not as significant for food. Moreover, adolescents could have saved beverages to share with family or friends later, which would have been more difficult with the food items. As mentioned above, we were not able to determine whether students traded their food and beverages.

We observed no significant price effect at baseline. This may be because some students may have considered the first voucher to be merely hypothetical; that is, they feared that on the following day, they would not in fact receive the food promised.

The individuals' peers had a large and statistically significant influence on their choices although less so in the case of sugar‐sweetened drinks. In this regard, we examined the contagious effects on obesity by exploring one of the underlying causes (food and beverage consumption), following Fortin and Yazbeck (2015). By contrast, other approaches have directly explored spillover effects by peers by examining their influence on BMI (Mora & Gil, 2013; Trogdon, Nonnemaker, & Pais, 2008). Nevertheless, the data obtained in our study are insufficient to determine whether the underlying process is affected by peers' actions or by the incentives offered (Angelucci, Prina, Royer, & Samek, 2015). Following the thinking in the mainstream literature, we assumed that peer influence was endogenous and that there was a reflection problem (Manski, 1993). However, our instrumental variable estimation showed the estimates of the peer effects to be unreliably large. It is possible that peers were more influential in the experimental group because the students interacted more strongly in the experimental classes than in the control classes.

Unlike some previous studies (Just & Price, 2013; List & Samek, 2015), we report evidence of incentivising unhealthy choices, that is, those arising from incentives promoting the consumption of unhealthy food and drinks. The results obtained reflect how teenagers react to promotions, such as those employed by supermarkets and corner stores, to counteract the impact of additional taxes on specific goods.

It is noteworthy that we identified some differences between groups of students in the impact of the nutrition workshop, particularly with regard to beverage choices: The difference is smaller or insignificant for male students and in public schools. These results provide new insights into designing policies and nutrition interventions in schools and suggest that some specific target groups (boys, public schools, and overweight children) require more research to design effective school interventions to change behaviours. In fact, it is a common finding in the literature on obesity interventions that boys do not respond well to such interventions (see, e.g., Brown & Summerbell, 2009; Stice, Shaw, & Marti, 2006). In our paper, we observed similar results; however, we also demonstrated that other personal characteristics make some difference in effectiveness as well.

The results of our study are useful for orienting nutritional and educational policies, suggesting that it may be more difficult to induce healthy choices in beverages than in food. In particular, sugared sodas are attractive to many schoolchildren, who tend to be resistant to nutritional advice. In this respect, male students and students attending public schools were not significantly affected by the nutrition workshop. Hence, alternative interventions or stricter regulations regarding labelling and advertising should be considered to reinforce the effectiveness of nutritional interventions in addition to more targeted programmes to change consumption patterns in adolescence. From the standpoint of public health, interventions aimed at restricting access to unhealthy beverages may be appropriate.

The educational intervention designed was effective although the magnitude of the effect was small. When unhealthy products were incentivised by means of price promotions (two for one), the positive effect of the workshop disappeared for certain groups of adolescents.

The study described has some limitations. First, although it was conducted in a large European city using a representative sample of schools, its external validity remains unknown. Policy advice derived from this study should not be mechanically applied to other contexts. Second, only short‐term reactions were measured. Long‐term changes in behaviour would most likely require ongoing nutritional advice, not merely a 1‐day workshop. Third, the influence of peers appears to be quite strong; however, technical difficulties in instrumenting this variable prevent us from making policy statements in this area. Fourth, and finally, our experimental design was intended to explore the impact of the nutrition workshop on the overall population of adolescents aged 12–13 years. However, the results obtained suggest that the intervention produces heterogeneous effects, varying among different groups of adolescents. Further investigation of this issue would require a more closely targeted experimental design.

## Supporting information

Appendix S1. Survey questionnaire
Appendix S2.
Table S1. Nutritional facts of the food and beverages of the experiment (information in grams)
Appendix S3. Estimated effect of price promotion at baseline
Table S2. Bivariate probit coefficients of price promotion at baseline (*t* = 0) and their standard errors corrected with bootstrap (250 replications)
Appendix S4. Peer effects
Table S3. Simultaneous estimation: instrumenting peers
