# Supplementary material for: Breakfast choice: An experiment combining a nutritional training workshop targeting adolescents and the promotion of unhealthy products
Source: Health Econ. 2017 Jul 25;27(2):306–19. doi: 10.1002/hec.3549 (PMC5901422; doi:10.1002/hec.3549)
Supplement: Supplementary file 1 — Appendix S1. Survey questionnaire Appendix S2. Table S1. Nutritional facts of the food and beverages of the experiment (information in grams) Appendix S3. Estimated effect of price promotion at baseline Table S2. Bivariate probit coefficients of price promotion at baseline (t = 0) and their standard errors corrected with bootstrap (250 replications) Appendix S4. Peer effects Table S3. Simultaneous estimation: instrumenting peers [file HEC-27-306-s001.docx]

**Appendix 1. Survey questionnaire**

**Scholar survey for habits 1^st^ ESO**

**Page. 1.- School and participant identification**

**------------------------------------------------------------------------------------**

**Quest.1.- School centre code**

**Quest.2.- Indicate your number on the class list**

**Page. 2.- Sociodemographic characteristics**

**------------------------------------------------------------------------------------**

**Quest.3.- Indicate your gender**

Boy Girl

**Quest.4.- Indicate your date of birth**

**Quest.5.- Were you born in the city of Barcelona?**

Yes No

**If you answered negatively, where were you born?**

Answer: _____________________________________________________________________________

**If you were born abroad, in which country?**

Answer: _____________________________________________________________________________

**If you were born abroad, in what year did you arrive in Catalonia?**

**Quest.6.- Where was your father born?**

Barcelona Catalonia Spain Europe Africa America Asia Don’t know

**Quest.7.- Where was your mother born?**

Barcelona Catalonia Spain Europe Africa America Asia Don’t know

**Quest.8.- Currently, who are you living with?**

Married parents Separated or single mother Separated father Separated mother and new partner

Separated father and new partner Widowed mother Widowed father With grandparents

With my tutors Other situations

**Quest.9.- Indicate father’s age**

**Quest.10.- Indicate mother’s age**

**Quest.11.- Number of siblings (not counting you)?**

**Quest.12.- Age ranking within siblings?**

**Page. 3.- Additional sociodemographic questions**

**------------------------------------------------------------------------------------**

**Quest.13.- Father’s occupation**

Retired Unemployed At home Working

**Quest.14.- Mother’s occupation**

Retired Unemployed At home Working

**Quest.15.- Father’s highest educational level**

No studies Primary Secondary Tertiary Don’t know

**Quest.16.- Mother’s highest educational level**

No studies Primary Secondary Tertiary Don’t know

**Quest.17.- State your level of agreement with the next sentences as they apply to you:**

|  | Completely  disagree | Disagree | Neither agree  nor disagree | Agree | Completely  agree |
| --- | --- | --- | --- | --- | --- |
| Tend to be lazy |  |  |  |  |  |
| Do thorough work |  |  |  |  |  |
| Make plans and follow through with them |  |  |  |  |  |
| Reliable worker |  |  |  |  |  |
| Tend to be disorganised |  |  |  |  |  |
| Easily distracted |  |  |  |  |  |
| Do things efficiently |  |  |  |  |  |
| Persevere until the task is finished |  |  |  |  |  |

**Page. 4.- Regular actions**

**------------------------------------------------------------------------------------**

**Quest.18.- Indicate your residence zip code**

**Quest.19.- How do you get to your school centre?**

Bus School bus Bike Walking Car Motorbike Train

**Quest.20.- Time to get to school**

1 to 10 minutes 10 to 20 minutes 20 to 40 minutes 40 to 60 minutes More than 1 h

**Quest.21.- Health status**

Very bad Bad Normal Good Very good

**Quest.22.- Indicate last term’s grades in the following subjects**

|  | Not Pass | Pass | Good | Very Good | Excellent |
| --- | --- | --- | --- | --- | --- |
| Mathematics |  |  |  |  |  |
| Catalan |  |  |  |  |  |
| Spanish |  |  |  |  |  |

**Quest.23.- Indicate your height**

**Quest.24.- Indicate your weight**

**Quest.25.- Write down the numbers of the classmates with whom you interact regularly**

**Quest.26.- Do you consider yourself the leader of your clique?**

Yes No

**Quest.27.- Do you receive pocket money regularly from your parents?**

Do not receive Ask when needed Get 1€-5€ Get 6€-10€ Get more than 10€

**Quest.28.- On what do you spend this pocket money? Several options can be marked**

Do not spend Candy Pastry (donuts, biscuits) Sugary drinks (Coca-Cola, Pepsi...)

Salty foods (popcorns, crisps) Ice-cream Clothing and accessories Games Journals

**Quest.29.- Mark all extracurricular activities in which you participate. Several options can be marked**

None Languages Sports Dance Chess Music Other

**Specify alternative activities:**

Answer: _____________________________________________________________________________

**Quest.30.- Indicate the amount of time you allocate to be with your friends doing the following activities each week:**

|  | Don’t go out | Less than  1 hour | 1 to 3 hours | 3 to 6 hours | More than  6 hours |
| --- | --- | --- | --- | --- | --- |
| We meet at someone else’s house. |  |  |  |  |  |
| Shopping |  |  |  |  |  |
| Drinking or eating |  |  |  |  |  |
| Going to the cinema |  |  |  |  |  |
| Homework |  |  |  |  |  |

**Page. 5.- Thanks for answering**

**------------------------------------------------------------------------------------**

**Quest.31.- Are you allergic to any food/drink?**

Yes No

**Type of allergy**

Gluten Fish Seafood Eggs Soya

Milk & lactose products Peanuts Nuts Sulphur dioxide and sulphites

**Select which food you want us to deliver to you as a reward for completing the survey (Images were shown)**

Regular voucher

Tuna sandwich plus sliced fruit Croissant Cupcakes Ham sandwich plus sliced fruit

Promoted voucher

Tuna sandwich plus sliced fruit 2 Croissants 2 Cupcakes Ham sandwich plus sliced fruit

**Select which drink you want us deliver to you as a reward for completing the survey (Images were shown)**

Regular voucher

Water Cola Milk Milk and sugared fruit juice

Promoted voucher

Water 2 Colas Milk 2 Milks and sugared fruit juices

**Page. 6.- You finished the survey:** Thanks for your cooperation!

**Appendix 2.**

**Table A1. Nutritional facts of the food and beverages of the experiment (information in grams)**

|  |  |  | Kcal | Proteins | Carbohydrates | Added sugars | Total lipids | Saturated fatty acids | Fibre |
| --- | --- | --- | --- | --- | --- | --- | --- | --- | --- |
| Drink | Healthy | Milk | 126.9 | 6.22 | 9.44 | 9.44 | 7.14 | 4.40 | 0 |
|  |  | Mineral water | 0 | 0 | 0 | 0 | 0 | 0 | 0 |
|  | Unhealthy | Cola | 129.9 | 0 | 32.47 | 32.47 | Traces | Traces | 0 |
|  |  | Sugared fruit juice | 58 | 0.8 | 13.2 | 13.2 | 0 | 0 | 1.2 |
| Food | Healthy | Tuna sandwich  (tuna, oil and bread) | 270.5 | 15.22 | 26.5 | 1.38 | 11.51 | 1.79 | 4.2 |
|  |  | Ham sandwich  (ham, oil and bread) | 307.27 | 11.43 | 26.5 | 1.38 | 17.27 | 3.77 | 4.2 |
|  |  | Sliced fruit | 91.14 | 1.18 | 20.54 | 0 | 0.34 | 0.11 | 3.025 |
|  | Unhealthy | Croissant | 222.6 | 4.12 | 30.25 | 4.12 | 9.46 | 5.45 | 1.21 |
|  |  | Cupcake | 212 | 3.36 | 21.95 | 10.56 | 12.31 | 6.82 | 0.55 |

**Appendix 3. Estimated effect of price promotion at baseline**

**Table A2.**

Bivariate probit coefficients of price promotion at baseline (t=0) and their standard errors corrected with bootstrap (250 replications).

|  | **No covariates** | **With covariates(*)** |
| --- | --- | --- |
| *Food promoted* | 0.042 (0.04) | 0.045 (0.04) |
| *Drink promoted* | 0.007 (0.05) | 0.009 (0.05) |

(*) The covariates include gender, being born in Barcelona, number of siblings, Body Mass Index, pocket money to spend, father and mother’s education, mother employed, monoparental household, dummy for having weekly money to spend and dummy for public school.

**Appendix 4. Peer effects**

The estimation of Eq. [2] by three-stage least squares, relating peer behaviour to the behaviour of friends-of-friends who are not friends of the respondent, which have been used as instruments (Table A2), showed that the nutrition workshop did not affect the choice of drink but did reduce the size and the significance of the effect on food choices (now only significant at 10%). The signs and sizes of the effects of the promotion vouchers remained unchanged. The instrumental variable estimation resulted in large variances of estimates, partially because the sample correlations between peer behaviour and the corresponding instruments are low (ranging from 0.10 for food at baseline to 0.36 for food at t=1). The instrumental variable estimation attributes a large effect to peers and overestimates the time trend compared with the baseline model described in Table 5, possibly because of the increasing correlation between the instrumental and the instrumented variables at t=1.

**Table A3. Simultaneous estimation: instrumenting peers**

|  | **Unhealthy**  **food choice** | **Unhealthy**  **beverage choice** |
| --- | --- | --- |
| Time effect (dummy for 2^nd^ voucher)  Workshop impact  Unhealthy peers impact  Two-for-one promotion at t=1 | -0.230 (0.06)***  -0.040 (0.02)*  0.576 (0.10)***  0.031 (0.02) | -0.537 (0.02)***  0.010 (0.01)  0.720 (0.16)***  0.038 (0.02)** |
| N  Chi^2^ (p-value) | 3,264  102.88 (0.00) | 3,264  37.29 (0.00) |

Note: ***, ** and * represent statistical significance at 1%, 5% and 10%. All results were obtained by seemingly unrelated equations estimation. Bootstrap analysis was performed, with 2,000 replicates. Standard errors were clustered at the school level.
